# A novel copper(I) complex induces ER-stress-mediated apoptosis and sensitizes B-acute lymphoblastic leukemia cells to chemotherapeutic agents

**DOI:** 10.18632/oncotarget.2027

**Published:** 2014-05-27

**Authors:** Roberta Bortolozzi, Giampietro Viola, Elena Porcù, Francesca Consolaro, Cristina Marzano, Maura Pellei, Valentina Gandin, Giuseppe Basso

**Affiliations:** ^1^ Department of Women's and Children's health, Oncohematology laboratory, University of Padova, Padova, Italy; ^2^ Department of Pharmaceutical and Pharmacological Sciences, University of Padova, Padova, Italy; ^3^ School of Science and Technology-Chemistry Division, Università di Camerino, Camerino, Macerata, Italy

**Keywords:** Acute lymphoblastic leukemia, ER stress, apoptosis, copper complexes

## Abstract

A phosphine copper(I) complex [Cu(thp)_4_][PF_6_] (CP) was recently identified as an efficient *in vitro* antitumor agent. In this study, we evaluated the antiproliferative activity of CP in leukemia cell lines finding a significant efficacy, especially against SEM and RS4;11 cells. Immunoblot analysis showed the activation of caspase-12 and caspase-9 and of the two effector caspase-3 and -7, suggesting that cell death occurred in a caspase-dependent manner. Interestingly we did not observe mitochondrial involvement in the process of cell death. Measures on semipurified proteasome from RS4;11 and SEM cell extracts demonstrated that chymotrypsin-, trypsin- and caspase-like activity decreased in the presence of CP. Moreover, we found an accumulation of ubiquitinated proteins and a remarkable increase of ER stress markers: GRP78, CHOP, and the spliced form of XBP1. Accordingly, the protein synthesis inhibitor cycloheximide significantly protected cancer cells from CP-induced cell death, suggesting that protein synthesis machinery was involved. In well agreement with results obtained on stabilized cell lines, CP induced ER-stress and apoptosis also in primary cells from B-acute lymphoblastic leukemia patients. Importantly, we showed that the combination of CP with some chemotherapeutic drugs displayed a good synergy that strongly affected the survival of both RS4;11 and SEM cells.

## INTRODUCTION

Many studies have shown that several chemotherapeutics drive cell death through the activation of ER stress. Thus, strategies that promote ER stress pro-death function or inhibit its prosurvival activity could be useful in cancer therapy [1, 2].

In response to ER stress and accumulation of unfolded proteins, cells activate a process known as Unfolding Protein Response (UPR). UPR integrates many signalling pathways to restore ER stability through the attenuation of protein synthesis and the upregulation of chaperones that facilitate protein folding. The main activator of UPR is the chaperon GRP78 which functions as a inhibitor of three ER transmembrane receptors: (PKR)-like ER kinase (PERK), inositol-requiring enzyme 1 (IRE1) and activating transcription factor 6 (ATF6) [3]. On misfolded proteins accumulation, GRP78 is required to target proteins for degradation and its release from ER membrane allows the activation of the three receptors. Initially, PERK is activated to block protein synthesis, followed by ATF6 to restore ER homeostasis and finally, IRE1 is activated with function of UPR mediator but also of ER stress-apoptosis inducer [4].

Misfolded proteins are ubiquitinated by the endoplasmic reticulum associated protein degradation system (ERAD) and degraded by proteasome thus reducing the toxic effects induced by protein aggregation [5]. However, alterations or pharmacological inhibition of ubiquitin proteasome system (UPS) prevent misfolded proteins degradation leading to ER stress condition [6]. Sustained and unsolved ER stress switches signalling from pro-survival UPR to cell death, via IRE-1. In turn, IRE-1 mediates the splicing of the transcription factor XBP-1 and the activation of the transcription factor CHOP (C/EBP-homologous protein), which ultimately drives the cell to apoptosis. The overload of ER-folding environment could also induces the apoptotic machinery through the activation of ER-resident caspase-12 [7, 8].

However, despite many studies, ER stress-induced cell death is not a well known process [2] and several mechanisms of programmed cell death, such as paraptosis, autophagy and apoptosis, have been described as consequence of prolonged ER stress [2, 9-11].

Recently, a novel phosphine copper(I) complex [Cu(thp)_4_][PF_6_] (CP) was found to have a marked *in vitro* antiproliferative activity against different human solid tumours, whereas it poorly affected non-tumour cells [12, 13]. The cytotoxic effect of CP in colon cancer cells has been correlated to the induction of a programmed non-apototic mechanism of cell death, called paraptosis or type III cell death [13]. Paraptosis lacks of apoptotic morphology, caspase-3 activation, DNA fragmentation and it is characterized by the massive presence of large vacuoles derived from endoplasmic reticulum, after the alteration of ER homeostasis [14]. Many studies show that copper complexes induce a disruption of proteasome-ER functional link through the inhibition of proteasome and the accumulation of misfolded proteins [15-17]. In particular, it has been demonstrated that, on colon cancer cells, the antiproliferative activity of CP is associated to functional suppression of the ubiquitin-proteasome pathway and to the induction of ER stress [13].

Up to now, very few works have described the effects of copper complexes on blood cancers and as concern CP only studies on solid tumors have been developed.

However, proteasome inhibitors such as Bortezomib, MG-132 and PS-341 are widely studied in haematological malignancy and seem very effective in inducing apoptosis. Moreover, many *in vivo* studies have demonstrated the efficacy of these compounds in combination with other chemotherapeutics. [18,19] Since the potential of proteasome inhibitors in leukemia treatment and the promising activity of CP on colon cancer cells, in this report we investigated CP effects on childhood leukemia cells.

We showed that CP had a strong growth inhibitory activity on several leukemia cell lines of different lineage and phenotype and it preferentially killed B-lymphoblastic leukemia cells. This cytotoxic activity was mediated by the induction of ER stress as a consequence of proteasome inhibition and accumulation of ubiquitinated proteins. Differently from what assessed in colon cancer cells, ER stress induced by CP triggered a caspase-dependent apoptotic program. More importantly, the association of CP with some chemotherapeutic drugs commonly used in therapy displayed a remarkable synergy that strongly affected the survival of both RS4;11 and SEM B-ALL cells.

## RESULTS

### CP induces growth inhibition in leukemia cell lines

[Cu(thp)_4_][PF_6_] (CP) was evaluated for its growth inhibition activity on a panel of twelve different human leukemia cell lines (five B-acute lymphoblastic leukemia, three T-acute lymphoblastic leukemia, three acute myeloid leukemia and one chronic myeloid leukemia). Cells were treated for 72 h with CP and cell viability was evaluated by MTT test. CP significantly inhibited leukemia cells growth with a GI_50_ ranging from 1.2 μM to 23 μM for myeloid phenotypes, between 3.9 μM and 16.7 μM for T-lymphoblastic phenotypes and from 0.9 μM to 4.2 μM for B-lymphoblastic cell lines (Table 1). In contrast, on both resting and PHA stimulated peripheral blood mononuclear cells (PBMC) from healthy donors, and on CD19^+^ isolated cells, the GI_50_ was generally higher than that on leukemia cells, suggesting that CP preferentially killed leukemia cells with a moderate selectivity toward B-lymphoblastic phenotype. Kumatori *et al.* [20] previously demonstrated that in malignant hematopoietic cells the expression of proteasome is at least 10 times higher that in lymphocytes and monocytes from healthy donors. This abnormal high expression of proteasomal proteins and mRNA seem to be correlated to the hyperproliferation of these cancer cells and to the sensitivity towards proteasome inhibitors [19,20].

**Table 1:** Cell growth inhibitory activity of CP in leukemia cell lines

Cell line	GI_50_ [μM]^a^
K562 (chronic myeloid leukemia)	23.2±1.0
HL60 (promyelocytic leulemia)	12.1±2.8
THP-1 (acute myeloid leukemia)	17.2±1
MV4;11 (acute myeloid leukemia)	1.2±0.2
Jurkat (T-acute lymphoblastic leukemia)	16.7±2.6
HSB-2 (T-acute lymphoblastic leukemia)	15.1±0.26
CCRF-CEM (T-acute lymphoblastic leukemia)	3.9±0.22
MHH-CALL2 (B-acute lymphoblastic leukemia)	1.9±0.1
RS4;11 (B-acute lymphoblastic leukemia)	0.3±0.01
REH (B-acute lymphoblastic leukemia)	4.2±0.2
SEM (B-acute lymphoblastic leukemia)	1.6±0.06
RCH-ACV (B-acute lymphoblastic leukemia)	0.9±0.2
Resting healthy PBMC	22.7± 2.9
PHA stimulated healthy PBMC	16.9±1.6

a GI50 = compound concentration required to inhibit tumor cell proliferation by 50%. Data are presented as the mean ± SE from the dose-response curves of three independent experiments.

Evaluated in the same experimental conditions, the inorganic salts CuCl and CuCl_2_ only induced a slight reduction of cell viability in all cell lines at concentration >250 μM (data not shown), pointing out the role of phosphine ligands in the cytotoxic action of CP.

Given the high efficacy of CP against B-leukemia phenotype and the bad prognosis of 4;11 translocated B-ALL, we further investigated the mechanism of action and the death pathways activated by CP in RS4;11 and SEM cells.

### Cellular uptake of CP is increased in B-leukemia cell lines

**FIGURE 1:**
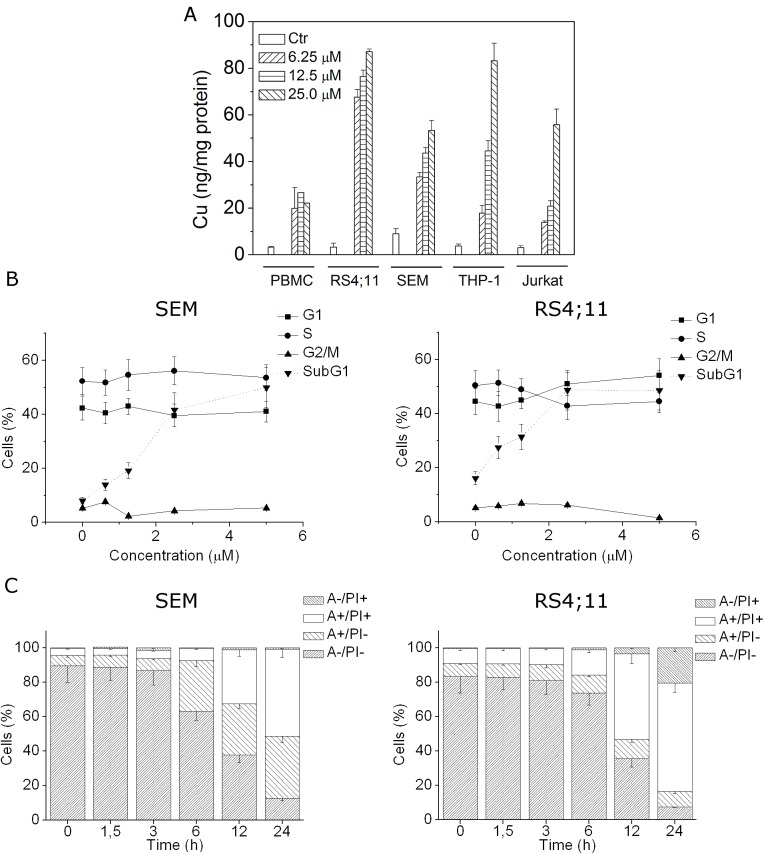
CP uptake and effects on cell cycle and apoptosis induction A) RS4;11, SEM, THP1, Jurkat cell lines and healthy PBMC were incubated with 6.25, 12.5 and 25 μM of CP for 24 h. The intracellular accumulation of CP was detected by GF-AAS analysis. B) SEM and RS4;11 cells were treated with the indicated concentrations of CP for 24 h, cell cycle distribution and Sub-G1 contents were analyzed by flow cytometry after cell staining with PI. The percentages of each phase of the cell cycle (G1, S, G2/M) were calculated on living cells whereas the percentages of cells with hypodiploid DNA content peak (subG1) was referred to cell population characterized by the appearance of subG1 peak. C) SEM and RS4;11 cells were incubated with 2.5 μM CP for the indicated times and analyzed by flow cytometry after double staining with Annexin-V-FITC and PI. Data are represented as mean ± S.E.M. of three independent experiments.

In an attempt to correlate the cytotoxic activity of CP with its cellular uptake, the intracellular copper content was evaluated in RS4;11, SEM, Jurkat and THP-1 cell lines and PBMC from healthy donors treated for 24 h with increasing concentrations of CP. The intracellular copper amount (Figure 1, panel A) was quantified by GF-AAS analysis (Graphite Furnace Atomic Absorption Spectrometry). Treatment of both leukemic cells with CP resulted in a marked increase of copper intracellular content in comparison with healthy PBMC. The higher uptake of CP in leukemic cells could be correlated with the higher cytotoxicity displayed by CP in this type of acute lymphoblastic leukaemia cell lines compared to healthy PBMC, supporting the better selectivity of CP towards cancer cells.

### Analysis of the CP effects on the cell cycle

The effects of different concentrations of CP on cell cycle progression were examined in RS4;11 and SEM cells after 24 h of treatment. Flow cytometric analyses revealed that CP did not affect cell cycle in these two cell lines (Figure 1, panel B) but more importantly, we observed a concentration-dependent increase of the cell population with a hypodiploid DNA content peak (subG1), which is usually considered as apoptotic cells, suggesting that CP may induce apoptosis (Figure 1, panel B).

### CP induces caspase-dependent cell death without cytochrome *c* release and mitochondrial membrane potential (MMP) depletion in B-leukemia cells

Many types of cell death are commonly induced in cancer cells: apoptosis, necrosis, autophagy, necroptosis and paraptosis. Recent studies showed that copper (I) and copper (II) complexes can induce paraptosis, a caspase-independent programmed cell death [15-17].

To characterize the mode of cell death induced by CP, we performed a cytofluorimetric analysis using PI and Annexin-V-FITC, which stain DNA and phosphatidylserine residues, respectively. After a time course of 1.5, 3, 6, 12 and 24 h of drug treatment (2.5 μM), SEM and RS4;11 cells were labeled with the two dyes and analyzed by flow cytometry. Starting from 6 h of treatment, we observed the appearance of significant levels of Annexin-V positive cells, that further increased at 12 and 24 h, in both cell lines, suggesting that apoptosis was induced by CP (Figure 1, panel C).

**FIGURE 2:**
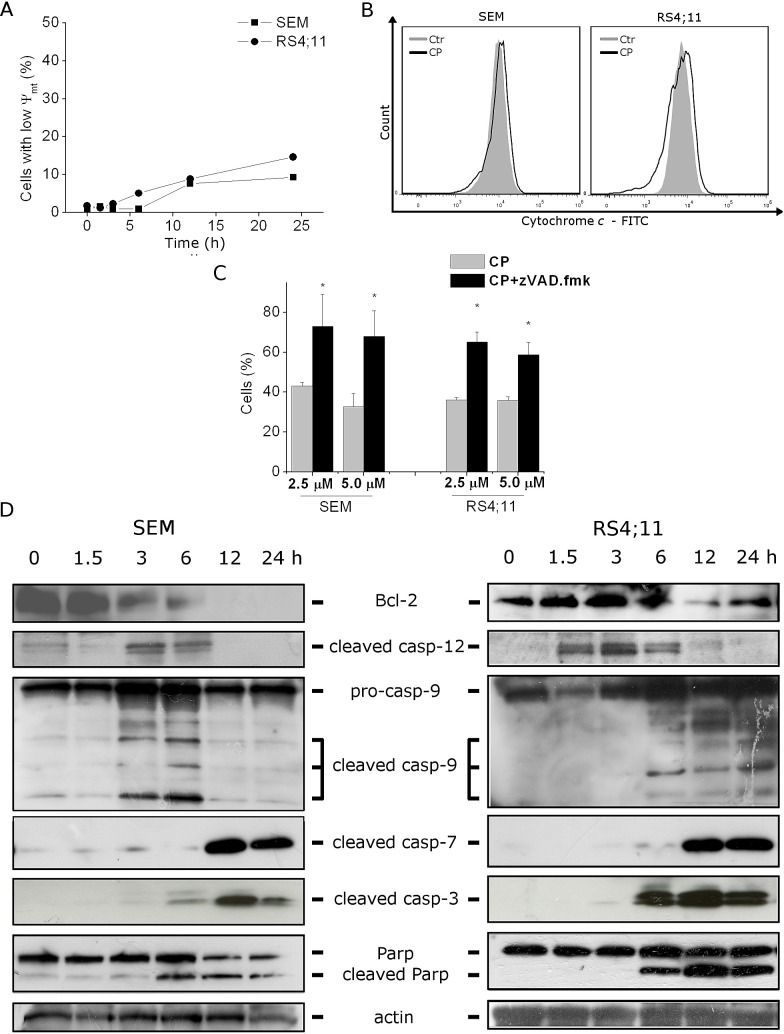
CP induces caspase-dependent apoptosis without mitochondria involvement (A) Assessment of mitochondrial membrane potential (ΔΨ_mt_) after treatment of RS4;11 and SEM cells with CP (2.5 μM) for the indicated times. Cells were stained with the fluorescent probe JC-1. Data are represented as mean ± S.E.M. of three independent experiments. (B) Flow cytometric analysis of RS4;11 and SEM cells treated with CP (2.5 μM) for 24 h showing cytochrome *c* labeled with a monoclonal antibody conjugated to FITC. Representative image of three experiments with similar results. (C) Cells were incubated with CP 2.5 μM and 5 μM in the presence or absence of the pan-caspase inhibitor z-VAD.fmk (100 μM). Cell viability was determined after 48 h by MTT assay. Data are represented as mean ± S.E.M. of three independent experiments performed in triplicate. *p<0.01 vs CP alone. (D) Western blot analysis of Bcl-2, cleaved caspase-12, caspase-9, cleaved caspase-7, caspase-3 and PARP after treatment of SEM and RS4;11 cells with CP (2.5 μM) for the indicated times. To confirm equal protein loading, each membrane was stripped and reprobed with anti-β-actin antibody.

To determine whether the apoptotic stimuli involved mitochondria alteration, we examined the mitochondrial transmembrane potential (ΔΨ_mt_) by means of the fluorescence dye JC-1. Cytofluorimetric analysis was performed on RS4;11 and SEM cells after different times of treatment, finding that the percentage of cells with low ΔΨ_mt_ slightly increased, only after 12 h (Figure 2, panel A). Moreover, further cytometric analysis did not reveal cytochrome *c* release, suggesting that MMP depletion was not involved in the apoptotic process (Figure 2, panel B).

We therefore evaluated whether the inhibition of caspases with the pan-caspase inhibitor z-VAD.fmk would prevent cell death. Our results assessed by MTT analysis showed that z-VAD.fmk significantly increased cell viability, suggesting that CP-induced cell death was mostly mediated by caspase-dependent apoptosis (Figure 2, panel C).

Concerning the involved caspases, immunoblot analysis (Figure 2, panel D) demonstrated the activation of caspase-9 and the cleavage of the two effector caspase-3 and -7 starting from 6 h of treatment, in agreement with the appearance of annexin-V positive cells described above. Accordingly, we also found, again just after 6 h of treatment, the cleavage of caspase-3 substrate PARP, a classical feature of apoptosis [21-23].

As a previous work, carried out by some of us, which showed ER stress involvement in the activation of cell death program induced by CP [13], we evaluated the activation of the ER-resident caspase-12. Many studies pointed out the central role exerted by caspase-12 in ER-stress induced apoptosis [7,8, 24,25]. Following its activation, caspase-12 migrates from ER to the cytosol and can directly process caspase-9, which in turn induces the activation of the apoptotic program, without cytochrome *c* involvement [8]. Starting from 1.5 h of CP treatment, in RS4;11 and from 3 h in SEM cell lines, we found the activation of caspase-12 prior to the activation of all the other caspases, suggesting that the process triggering apoptosis could start from an ER impairment.

Furthermore, a decrease in the expression of the anti-apoptotic protein Bcl-2 was detectable at least after 3 h of treatment and this correlates with an early commitment to apoptosis (Figure 2, panel D).

### CP induces the impairment of Ubiquitin-Proteasome System

**FIGURE 3:**
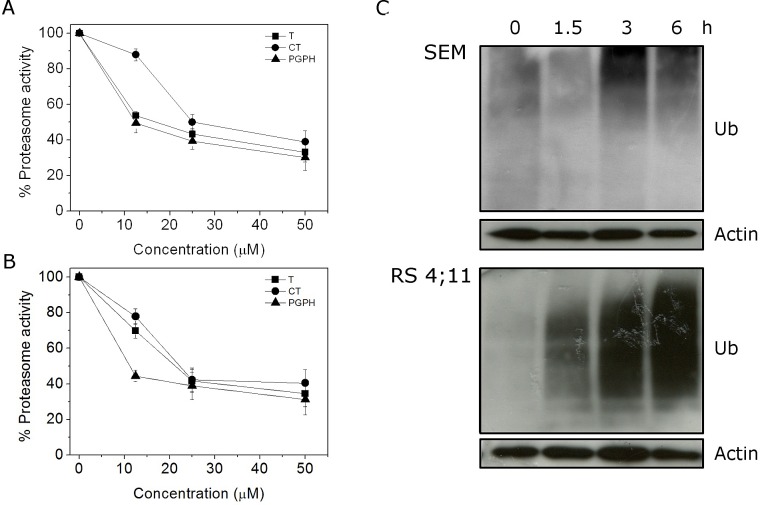
CP treatment affects Ubiquitin Proteasome System (UPS) 26S proteasome (100 μg) semipurified from (A) SEM and (B) RS4;11 cells was incubated for 60 min at 37 °C with CP at different concentrations. Chymotrypsin (CT), trypsin (T) and peptidyl glutamyl peptide hydrolyzing (PGPH) catalytic activity were measured fluorimetrically (excitation 370, nm emission 460) by following the release of free AMC from synthetic substrates N-Suc-Leu-Leu-Val-Tyr-AMC, Boc-Gln-Ala-Arg-AMC and Z-Leu-Leu-Glu-AMC, specific for CT, T and PGPH activity, respectively. (C) Cells were incubated with CP 2.5 μM for the indicated times and then western blot analyses were performed to evaluate Ubiquitin accumulation.

Previous observations indicated that in colon cancer cells CP may induce functional suppression of the ubiquitin–proteasome pathway inhibiting all the proteolytic activity of the proteasome [13]. The 26S proteasome is a multicatalytic protease responsible for the regulated intracellular protein degradation. Its function is mediated by three main catalytic activities i) chymotrypsin like (CT) ii) Trypsin-like (T) and iii) peptidyl glutamyl peptide hydrolyzing (PGPH). To evaluate the effect of CP on the proteasome activity in leukemia cells, we tested increasing concentrations of the compound on semipurified 26S proteasome extract from RS4;11 and SEM cells, and measured the functioning of each individual proteolytic activity under cell-free condition. Both in RS4;11 and SEM cells (Figure 3, panels A and B), CP inhibited all the proteolytic activities with IC_50_ values of about 15, 12 and 16 μM in SEM and 18, 11 and 10 μM in RS4;11 for CT, T and PGPH activity, respectively.

In normal conditions, misfolded proteins in the ER are driven to the cytoplasm, where upon ubiquitination, are degraded by the proteasome system. Proteasome inhibitors have been shown to induce an accumulation of ubiquitinated proteins in the cytoplasm and ER stress.[1] To assess if CP worked as other proteasome inhibitors, we evaluated the accumulation of ubiquitin after 1.5, 3, and 6 h of treatment. Immunoblot analysis (Figure 3, panel C) revealed that CP induced the accumulation of ubiquitinated proteins starting from 1.5 h in SEM and 3 h in RS4;11 cells.

### CP induces ER stress in RS4;11 and SEM cells lines

To evaluate if CP effects on proteasome activity lead to ER stress, we evaluated the expression of ER stress marker GRP78, CHOP and the splicing of XBP1 in CP-treated RS4;11 and SEM cells [26,27].

**FIGURE 4:**
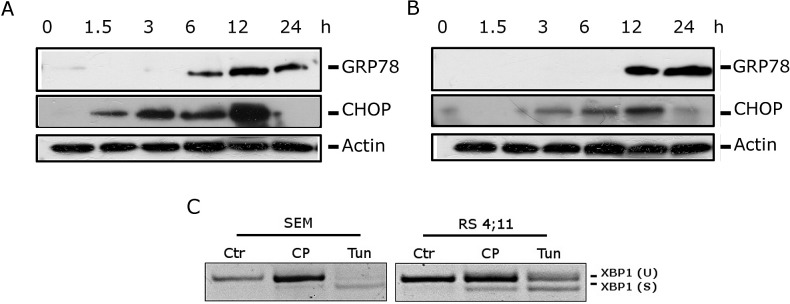
CP induces ER stress Western Blot analysis showing expression of GRP78 and CHOP in SEM (A) and in RS4;11 (B) cells. Cells were treated with CP 2.5 μM for the indicated times. (C) PCR analysis showing the induction of XBP1 splicing in SEM and RS4;11 cells. Cells were treated with CP (2.5 μM) or Tunicamycin (2 μg/ml) as positive control for 12 h.

Western blot analyses demonstrated a remarkable increase of GRP78 expression after 6 h of treatment in SEM and after 12 h in RS4;11(Figure 4, panels A and B). Moreover we found an increase in CHOP expression starting from 3 h of CP treatment. Interestingly, the CHOP increased expression was detectable earlier than the induction of GRP78. Whilst GRP78, due to its chaperon activity, exerts a protective function, CHOP promotes ER-stress cell death [28,29], thus the rapid activation of pro-death signals and the late activation of pro-survival chaperone GRP78 could explain the commitment toward apoptosis induced by CP. Moreover, in unresolvable stress conditions, an increased CHOP expression has been correlated to the induction of the expression of many apoptotic genes and to the down-regulation prosurvival genes, such as Bcl-2 [30]. Indeed, as showed above, we found a decrease in Bcl-2 protein (Figure 2, panel D) following CHOP activation. In addition we observed that CP induced XBP1 splicing in both cell lines (Figure 4, panel C).

### CP-induced apoptosis is mediated by ER stress

To investigate if proteasome inhibition and ER stress could be responsible for CP-induced cell death, we assayed apoptosis and ER stress markers in the presence of cycloheximide (CHX), an inhibitor of protein synthesis that antagonizes ER-stress agents [24]. The inhibition of protein synthesis reduced the accumulation of unfolded proteins in the ER and therefore ER-stress induced apoptosis.

**FIGURE 5:**
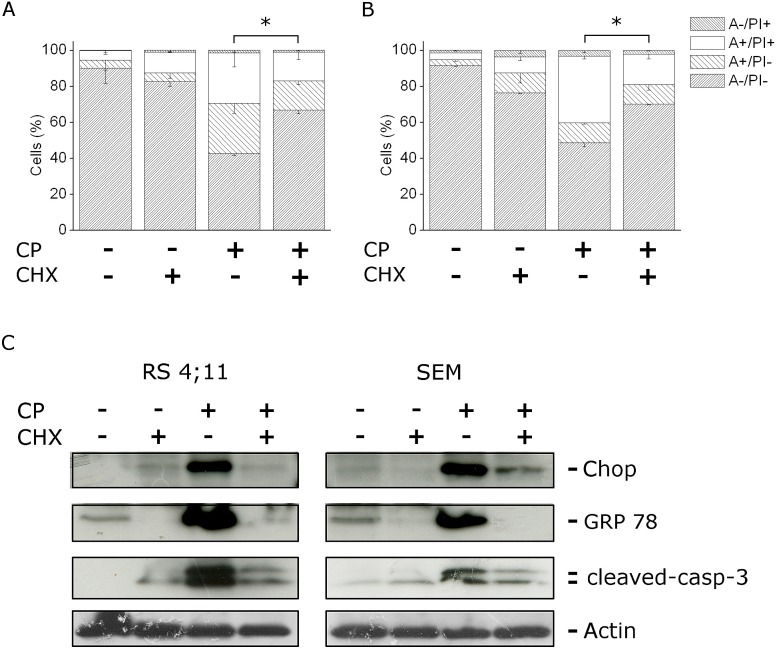
CP induces ER stress mediated apoptosis SEM (A) and RS4;11 (B) cells were incubated with CP (2.5 μM) in the presence or absence of cycloheximide (CHX) for 12 h, at the concentration of 2 μg/ml. Cells were analyzed by flow cytometry after double staining with Annexin-V-FITC and PI. Data are represented as mean ± S.E.M of three independent experiments. *p<0.05. (B) Western blot analysis showing the expression of GRP78 and CHOP after treatment of RS4;11 and SEM cells with CP (2.5 μM) for 12 h in the presence or absence of CHX (2 μg/ml).

As shown in Figure 5, panels A and B, the Annexin-V/PI assay revealed that CHX effected a significant protection from CP induced cell death and a significant reduction of caspase-3 cleavage, in both RS4;11 and SEM cells. Furthermore, CHX induced a remarkable reduction of GRP78 and CHOP expression, induced after 12 h of treatment with CP, when CHOP and GRP78 were highly expressed (Figure 5, panel C). These data suggested that protein synthesis machinery was involved in the mechanism of cell death and supported the hypothesis that CP-induced apoptosis is activated by ER-stress signals.

### CP induces ER-stress and apoptosis in B-acute lymphoblastic leukemia primary cultures

**FIGURE 6:**
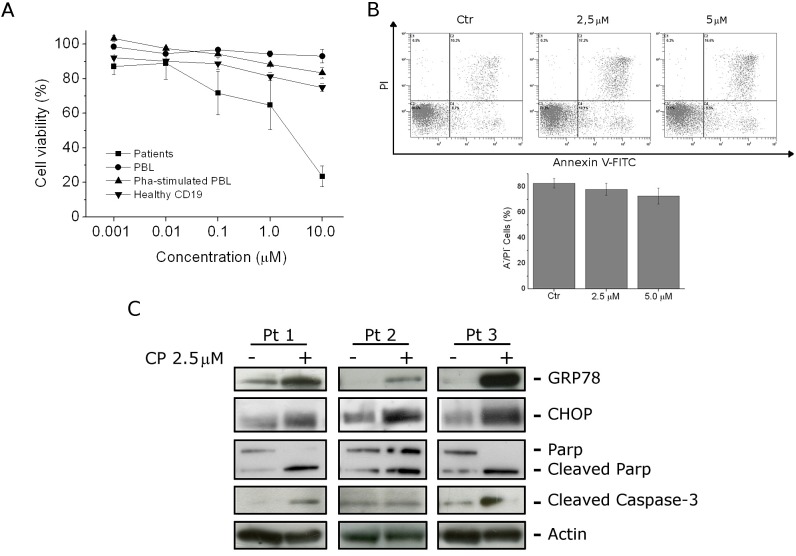
CP effects on B-ALL primary cultures (A) Blast cells from six B-ALL patients were incubated for 24 h with different concentrations of CP. For the sake of comparison CD19^+^ cells from peripheral blood of healthy donors, and PBMC were also incubated with CP in the presence or absence of PHA. Cell viability were assayed by MTT analysis. Data are represented as mean ± S.E.M of four independent experiments. (B) Bone marrow cells from healthy donors were treated for 24 h with CP (2.5 μM and 5 μM). Cell viability was evaluated on the whole population by flow cytometry after staining the cells with Annexin-V-FITC and PI. are represented as mean ± S.E.M of 3 independent experiments. (C) Western Blot analysis of GRP78 and CHOP expression, PARP cleavage and caspase-3 activation, on protein extracts of blast cells from B-ALL patients treated with CP (2.5 μM) for 12 h.

CP growth inhibitory activity was measured on primary cultures derived from pediatric patients affected by B-acute lymphoblastic leukemia. After 24 h of treatment, MTT analysis revealed a GI_50_ of 2.3 μM in B-acute lymphoblastic leukemia patients while GI_50_ was significantly higher in healthy lymphocytes. In particular, in resting and in PHA-stimulated PBMC, and in CD19^+^ cells GI_50_ was higher than 10 μM confirming that CP preferentially affects leukemia cells. This trend was also supported by the finding that CP treatment of bone marrows cells, derived from healthy donors, did not affect the viability of both lymphocyte and monocyte populations (Figure 6, panel A and B).

On the other hand, treatment of patient cells with CP (2.5 μM) for 12 h induced caspase-3 activation and PARP cleavage confirming the onset of caspase-dependent apoptosis also on B-lymphoblastic leukemia primary cells (Figure 6, panel C). Moreover, monitoring GRP-78 and CHOP expression in primary cultures, a significant increase in the expression of both these markers was found, indicating ER-stress response, as observed in RS 4;11 and SEM cell lines (Figure 6, panel C).

### CP synergizes with drugs used in B-ALL chemotherapy

Preclinical studies demonstrated that proteasome inhibitors are highly effective in leukemia in combination with other commonly used chemotherapeutics, and phase I trial on ALL of Bortezomib combined with chemotherapy showed significant results [31].

We investigated the effect of CP in RS4;11 and SEM cell lines in combination with different chemotherapeutic drugs commonly used in B-ALL treatment, to assess possible synergistic interactions.

SEM and RS4;11 cell lines were exposed to dexamethasone (Dex), daunorubicine (Dauno), cytarabine (Ara-C) and vincristine (Vcr) in the presence or absence of CP, at fixed combination ratios. Table 2 summarizes the results of drug combinations analysed by Chou and Talalay method [32]. In both cell lines, CP increased the antiproliferative effects of all the drugs in a synergistic way, in particular at high effect levels (GI_75_, GI_90_) that represent the most therapeutically relevant conditions for cancer therapy [33]. We found a very strong synergism (CI<0.1) in the case of CP-Dex and CP-Vcr combination in both cell lines, and by combining CP with CP-AraC in SEM cells (Table 2). It is important to note that the effect of CP promoted a synergistic cytotoxic effect when administered with chemotherapeutic drugs endowed with different mechanisms of action, suggesting that the induction of ER-stress, could be potentially useful in clinical therapy to optimize the efficacy of existing therapies for B-leukemias.

**Table 2:** Effects of the combination of CP with chemotherapeutic agents

Cells	Drug combination	GI_50_ (nM)^a^	CI value at		
			GI_50_	GI_75_	GI_90_		
SEM	Dex	643±44.2			
	Dex+CP (1:10)^b^	113±8.8	0.3	0.2	0.13
	Dauno	96±14.1			
	Dauno+CP (1:10)^b^	28.7±2.7	0.69	0.61	0.54
	AraC	22.3±1.8			
	AraC+CP (1:1)^b^	19.3±0.7	0.065	0.08	0.02
	Vcr	13.1±1.2			
	Vcr+CP (1:100)^b^	2.1±0.3	0.06	0.04	0.05
RS4;11	Dex	0.12±0.025			
	Dex+CP (1:10)^b^	0.05±0.01	16.8	0.55	0.3
	Dauno	19.2±1.9			
	Dauno+CP (1:10)^b^	4.8±0.36	0.47	0.31	0.21
	AraC	21±7.1			
	AraC+CP (1:1)^b^	5.9±0.9	0.93	0.61	0.4
	Vcr	0.071±0.042			
	Vcr+CP (1:100)^b^	0.016±0.004	1.5	0.06	0.15

Abbreviations: Ara-C, cytarabine; CI, combination index; Dex, dexamethasone; Dauno, daunorubicin; Vcr, vincristine. Synergy, additivity and antagonism are defined by a CI<1, CI=1 or CI>1, respectively.

a compound concentration required to inhibit tumor cell proliferation by 50%. Data are presented as the mean ± SE from the dose-response curves of three independent experiments.

b Molar combination ratios

## DISCUSSION

CP is a monocationic copper (I) complex that was found to be an effective antiproliferative agent in colon cancer cells through the impairment of ubiquitin/proteasome system and the activation of paraptosis, an alternative form of programmed cell death characterized by cytoplasmic vacuolization, mitochondrial swelling and the absence of caspase activation [10].

In this study, we demonstrated that CP is effective also in leukemic cells with a growth inhibitory activity comparable and in some cases even higher than that observed in colorectal cancer cells. The cytotoxic activity on healthy lymphocytes was 5-10 fold lower than B-leukemia cells, suggesting a preferential cytotoxicity versus this lineage of neoplastic cells. This finding may be related to the higher CP intracellular content observed in RS4;11 and SEM B-cells compared to healthy PBMC.

Herein we demonstrated that, in RS4;11 and SEM B-leukemia cell lines, CP induced the accumulation of ubiquitinated proteins and ER stress through the inhibition of proteasome system and suppression of protein degradation. The inhibition of protein synthesis by CHX protected cells from the effect deriving from CP treatment, supporting the hypothesis of UPS impairment and ER stress induced cell death.

Besides activating apoptotic pathways, ER stress is also known to trigger survival signals by increasing the expression of pro-survival UPR components such as the chaperon GRP78. In this context, it has recently reported that GRP78 is highly expressed in response to ER stress stimuli and may play an important role in cell survival of leukemic cells [34-36].

Our data revealed, upon treatment with CP, an early activation of the proapoptotic transcription factor CHOP, a well-known marker of ER-stress, and this activation closely correlated with the appearance of Annexin-V positive cells, the activation of caspases and downregulation of the pro-survival protein Bcl-2. It has been shown that prolonged high expression levels of CHOP led ER-stress to a proapototic cell fate, despite the activation of the pro-survival chaperon GRP78 [28,29]. The increase of both CHOP and GRP78 consequent to CP treatment suggests that CP led a sustained activation of ER-stress, that was able to overcome GRP78 protective mechanisms that occurred later than CHOP activation, thus promoting the induction of apoptotic cascade.

Differently from colon cancer cells, where the activation of ER stress by CP led to paraptosis, in B-lymphoblastic leukemia cells it triggered a caspase-dependent apoptosis as demonstrated by the significant increase of cell viability in the presence of the pancaspase inhibitor z-VAD.fmk. Moreover, the cleavage of both caspase-9 and caspase-3 as well as caspase-7 unequivocally confirmed the activation of the apoptotic process. Interestingly, CP treatment only slightly increased mitochondrial depolarization and didn't cause cytochrome *c* release, suggesting that the mitochondrial pathway was not involved or, at least it did not play a major role. It is worth to note that mitochondria-independent cell death has been described to mediate apoptosis in response to ER stress induced by different stimuli [37,38].

The accumulation of ubiquitinated proteins and the persistent ER stress primed death signals from the ER stress sensors and the activation of the ER-resident caspase-12. The discovery of an ER-localized caspase-12 has brought into question the mechanism by which ER stress activates caspases and trigger apoptosis. *In vitro* active caspase-12 is able to directly cleave caspase-9 which subsequentially activates caspase-3, potentially eliminating the requirements of the mitochondria and the apoptosome to perform ER-stress induced apoptosis [7,8]. This mechanism of action has been observed in C2C12 cells treated with known ER-stress inducers such as thapsigargin and tunicamycin where caspase-9 could be processed by caspase-12 in an Apaf-1/cytochrome *c* independent manner [8]. Although this mechanism was observed in murine myoblasts, a large number of studies carried out in human cells have indicated that caspase-12 may be activated by ER stress consequent to different causes, including anticancer drugs [39-44]. Consistent with these observations, our results showing the early cleavage of caspase-12 prior to caspase-9 and caspase-3 activation, suggested the direct involvement of caspase-12 in initiating the apoptotic program.

The strong ability of CP to induce cell growth inhibition and apoptosis response was also confirmed in *ex-vivo* B-leukemia cell culture. The activation of ER-stress markers suggested a link between cell death and unfolded protein response and ER-stress overload also in primary cells. Another important aspect of our study, is that CP exhibits a strong significative effect in killing leukemic cells in association with many of the chemotherapeutics currently used in therapy. Leukemic cells and in general cancer cells are characterized by enhanced activation of pro-survival pathways that may protect cells from stress induced by chemotherapeutic agents. It is possible that CP sensitizes cancer cells by an overload of ER stress condition able to improve leukemia cell death in a synergistic way. It is worthwhile to note that this effect appears with a large number of drugs currently used that work with different mechanism of action, such as microtubule inhibitors, DNA damaging agent and antimetabolites. These results suggest that CP could increase the effects of conventional chemotherapy for B-ALL and pointed towards the potential clinical utility of ER-stress inducers, although further studies are needed to better understand the molecular mechanism(s) involved in the synergistic effect.

## MATERIALS AND METHODS

### Chemicals

[Cu(thp)_4_][PF_6_], abbreviated CP was synthesize as previously described[12]. N-[(benzyloxy)carbonyl]-L-Val-Ala-DL-Asp-fluoromethylketone (z-VAD.fmk), tunicamycin and cycloheximide (CHX) were purchased from Sigma-Aldrich (Italy).

### Cell Lines and Growth Inhibition Assay

The human leukemia cell lines RS 4;11, SEM, REH, RCH-ACV, MHH-CALL2, Jurkat, CCRF-CEM, HSB2, HL-60, K562, THP-1, MV4;11 were purchased from the American Type Culture Collection. Cells were cultured in RPMI 1640 (Life Technologies, Italy) supplemented with 10% fetal bovine serum (FBS), glutamine (2 mM; Life Technologies, Italy), penicillin (100 U/ml; Life Technologies, Italy) and streptomycin (100 μg/ml; Life Technologies, Italy), and maintained at 37°C in a humidified atmosphere with 5% CO_2_. The cytotoxic activity of CP was determined using a standard 3-[4,5-dimethylthiazol-2-yl]-2,5-diphenyltetrazodium bromide (MTT) based colorimetric assay (Sigma-Aldrich, Italy). Briefly, cells were seeded at a density of 2×10^4^ cells/well in 96-well microtiter plates. After 24 h, cells were exposed to the test compound. After different times, cell survival was determined by the addition of an MTT solution as previously described [45].

### Primary leukemia cell cultures

B-ALL patient samples were obtained after informed consent following the tenets of the Declaration of Helsinki. The study was approved by the Ethical Committee board of the University of Padova, the Padova Academic Hospital and the Italian Association of Pediatric Onco-Hematology (AIEOP). Diagnosis was made according to standard cytomorphology, cytochemistry and immunophenotypic criteria [46]. All analyzed B-ALL samples were obtained at the time of diagnosis before treatment, after Lymphoprep (Fresenius KABI, Norge AS) separation of mononuclear cells. The percentage of CD19^+^ cells ranged from 80% to 95%.

Peripheral blood mononuclear cells (PBMC) and bone marrow cells from healthy donors were obtained by separation on Lymphoprep (Fresenius KABI, Norge AS) gradient. After extensive washing, cells were resuspended (1.0 × 10^6^ cells/ml) in RPMI1640 with 10% fetal bovine serum and incubated overnight in 96-well tissue culture microtiter plate. For cytotoxicity evaluations in proliferating PBMC cultures, non-adherent cells were resuspended in growth medium, containing 2.5 μg/ml phytohematoglutinin (PHA) (Irvine Scientific). To isolate B-lymphocyte, PBMC obtained by Lymphoprep (Fresenius KABI, Norge AS) separation were labeled with anti-CD19-APC (BD Biosciences, Italy) and collected by cell sorting.

### Externalization of Phosphatidylserine (PS)

Surface exposure of PS by apoptotic cells was measured by flow cytometry with a Coulter Cytomics FC500 (Beckmann Coulter, USA) by adding Annexin V-FITC and propidium iodide (PI) to cells according to the manufacturer's instructions (Annexin-V Fluos, Roche Diagnostic, Italy).

### Analysis of Cell Cycle Distribution

5×10^5^ RS4;11 and SEM cells in exponential growth were treated with different concentrations of CP for different times. After the incubation, cells were collected, centrifuged and fixed with ice-cold ethanol (70%) and stained with PI as described previously [45]. DNA histograms were analyzed using Multicycle for Windows (Phoenix Flow Systems)

### Assessment of Mitochondrial Changes and Release of Cytochrome c

The mitochondrial membrane potential was measured with the lipophilic cation 5,5',6,6'-tetrachlo-1,1',3,3'-tetraethylbenzimidazolyl-carbocyanine (JC-1) (Life Technologies, Italy) as previously described [45]. Cytochrome *c* release was analyzed by flow cytometry using a commercial kit (Innocyte flow cytometric cytochrome *c* release kit, Calbiochem, Italy) following the manufacturer's instructions.

### Proteasome activity on purified cell extracts

RS4;11 and SEM cells were homogenized in a lysis buffer (50 mM Tris-HCl, pH 7.5, 250 mM sucrose, 5 mM MgCl2, 1 mM DTT, 0.5 mM EDTA) and ultracentrifuged for 2 h at 300,000 g. Total protein content was estimated with BioRad protein assay (BioRad) and 100 μg of protein aliquots were incubated for 60 min at 37°C with with CP at different concentrations. Afterwards, fluorogenic proteasome substrates (N-Suc-Leu-Leu-Val-Tyr-7-amido-4-methylcoumarin (AMC) for chymotripsin (CT), Boc-Gln-Ala-Arg-AMC for tripsin (T) and Z-Leu-Leu-Glu-AMC for peptidyl glutamyl peptide hydrolyzing (PGPH) were added and the substrate hydrolysis was measured after 30 min by monitoring the release of Tyr-7-amido-4-methylcoumarin (AMC) using a Varian Cary Eclipse (Varian) spectrofluorometer (excitation at 370 nm, emission at 460 nm).

### Cellular uptake

RS4;11, SEM, Jurkat, THP-1 and PBMC cells (7×10^5^) were seeded in 25 cm^2^ flasks in growth medium. After 24 h, the cells were incubated for further 24 h with 6.25, 12.5 and 25 μM of CP. Cells were washed with PBS and harvested. Samples were subjected to three freezing/thawing cycles at −80 °C, and then vigorously vortexed. Aliquots were removed for the determination of protein content by the BioRad protein assay (BioRad). The samples were added with 1 ml highly pure nitric acid (Cu: ≤0.005 μg/kg, TraceSELECT® Ultra, Sigma Chemical Co.) and transferred into a microwave teflon vessel. Subsequently, samples were submitted to standard procedure using a speed wave MWS-3 Berghof instrument (Eningen, Germany). After cooling, each mineralized sample was analyzed for copper amount by using a Varian AA Duo graphite furnace atomic absorption spectrometer (Varian, Palo Alto, CA; USA) at 324 nm. The calibration curve was obtained using known concentrations of standard solutions purchased from Sigma Chemical Co.

### Western Blot Analysis

Cells were treated with CP and, after different times, were collected, centrifuged and washed with ice cold phosphate-buffered saline (PBS). The pellet was then resuspended in lysis buffer as described [47]. The protein concentration in the supernatant was determined using the BCA protein assay (Pierce, Italy). Equal amounts of protein (10 μg) were resolved using SDS-PAGE gel electrophoresis (*Criterion precast* Tris-HCl *gel, BioRad*, Italy) and transferred to PVDF Hybond-p membranes (GE Healthcare, Italy). Membranes were blocked with 2% ECL-Blocking Solution (GE Healthcare, Italy) for 2 hours at room temperature. Membranes were then incubated with primary antibodies against Bcl-2, PARP, procaspase-9, cleaved caspase-7, GRP78, (Cell Signaling, Italy), cleaved caspase-12 (Abcam, UK), β-actin (Sigma Aldrich, Italy), and cleaved caspase-3 (Novus Biologicals, Italy) overnight. Membranes were then incubated with peroxidase-conjugated secondary antibodies (Invitrogen, Italy) for 60 min. All membranes were visualized using ECL Select (GE Healthcare, Italy) and exposed to Hyperfilm MP (GE Healthcare, Italy). To ensure equal protein loading, each membrane was stripped and reprobed with anti-β-actin antibody.

### XBP1 Splicing

Cells were treated with CP and Tunicamycin as positive control, collected, centrifuged and washed with PBS. Total RNA was extracted from cells using Trizol (Life Technologies, Italy) as previously described [47]. Primer pair used to amplified XBP1 isoforms: forward 5'-TTACGAGAGAAAACTCATGGCC-3', reverse 5'-GGGTCCAAGTTGTCCAGAATGC-3'. To separate isoforms, PCR products were run in 5% agarose gel.

### Combined drugs analysis

To test potential synergistic, additive, or antagonistic effects of the combination of CP and drugs commonly used in B-ALL treatment, we performed MTT experiments as follows.

SEM, and RS4;11 cells were treated for 48 hours with one of the most used chemotherapeutic agents: cytarabine (Aractyn, Pfizer), daunorubicin (Pfizer), vincristine (Pfizer), or dexamethasone (Sigma-Aldrich). CP was also added to each drug solution, at fixed combination ratios. Cell viability was determined after 48 hours of treatment by MTT test as described above. To determine the synergistic, additive, or antagonistic effects of the drug combinations, we used CalcuSyn software (version 2.0, Biosoft, Cambridge, United Kingdom) based on the method of the combination index (CI) described by Chou and Talalay [32]. Synergy, additivity and antagonism were defined by a CI<1, CI=1, or CI>1, respectively.

### Statistical Analysis

Unless indicated otherwise, results are presented as mean ± S.E.M. The differences between different treatments were analyzed using the two-sided Student's t test. P values lower than 0.05 were considered significant.
